# Auto-Tandem Catalysis in Ionic Liquids: Synthesis of 2-Oxazolidinones by Palladium-Catalyzed Oxidative Carbonylation of Propargylic Amines in EmimEtSO_4_

**DOI:** 10.3390/molecules21070897

**Published:** 2016-07-08

**Authors:** Raffaella Mancuso, Asif Maner, Ida Ziccarelli, Christian Pomelli, Cinzia Chiappe, Nicola Della Ca’, Lucia Veltri, Bartolo Gabriele

**Affiliations:** 1 Laboratory of Industrial and Synthetic Organic Chemistry (LISOC), Department of Chemistry and Chemical Technologies, University of Calabria, Via Pietro Bucci 12/C, 87036 Arcavacata di Rende (CS), Italy; asifncl@gmail.com (A.M.); idaziccarelli@gmail.com (I.Z.); lucia.veltri@unical.it (L.V.); 2 Department of Pharmacy, University of Pisa, Via Bonanno 33, 56126 Pisa, Italy; christian.pomelli@gmail.com (C.P.); cinzia.chiappe@farm.unipi.it (C.C.); 3 Department of Chemistry, University of Parma, Parco Area delle Scienze 17/A, 43124 Parma, Italy; nicola.dellaca@unipr.it

**Keywords:** carbonylation, cascade catalysis, oxazolidinones, palladium

## Abstract

A convenient carbonylative approach to 2-oxazolidinone derivatives carried out using an ionic liquid (1-ethyl-3-methylimidazolium ethyl sulfate, EmimEtSO_4_) as the solvent is presented. It is based on the sequential concatenation of two catalytic cycles, both catalyzed by the same metal species (auto-tandem catalysis): the first cycle corresponds to the oxidative monoaminocarbonylation of the triple bond of propargylic amines to give the corresponding 2-ynamide intermediates, while the second one involves the cyclocarbonylation of the latter to yield 2-(2-oxooxazolidin-5-ylidene)-acetamides. Reactions are carried out using a simple catalytic system consisting of PdI_2_ in conjunction with an excess of KI, and the catalyst/solvent system could be recycled several times without appreciable loss of activity after extraction of the organic product with Et_2_O.

## 1. Introduction

**Scheme 1:**
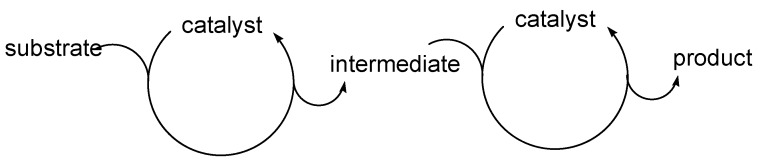
The concept of “auto-tandem catalysis; the same catalyst promotes the two concatenated catalytic cycles.

Cascade catalysis, in which a catalytic cycle is concatenated to another eventually leading to the final product, is one of the most exciting areas of modern catalysis [1,2,3,4,5,6,7,8,9,10]. Although rather frequent in biological systems [11], where processes may be sequentially catalyzed by different enzymes, it is still relatively rare in chemical transformations, where they usually involve two concatenated cycles. A particularly interesting case, commonly referred as “auto-tandem catalysis” [9], occurs when the same catalytic system is able to catalyze *both* the concatenated cycles, as shown in Scheme 1.

In this work, we report on the direct synthesis of 2-oxazolidinone derivatives through an auto-tandem catalysis process consisting of the concatenation of two carbonylative catalytic cycles, both catalyzed by the same catalytic system (PdI_2_ in conjunction with an excess of KI), performed in the ionic liquid 1-ethyl-3-methylimidazolium ethyl sulfate (EmimEtSO_4_) as an unconventional solvent. 

## 2. Result and Discussion

**Scheme 2:**
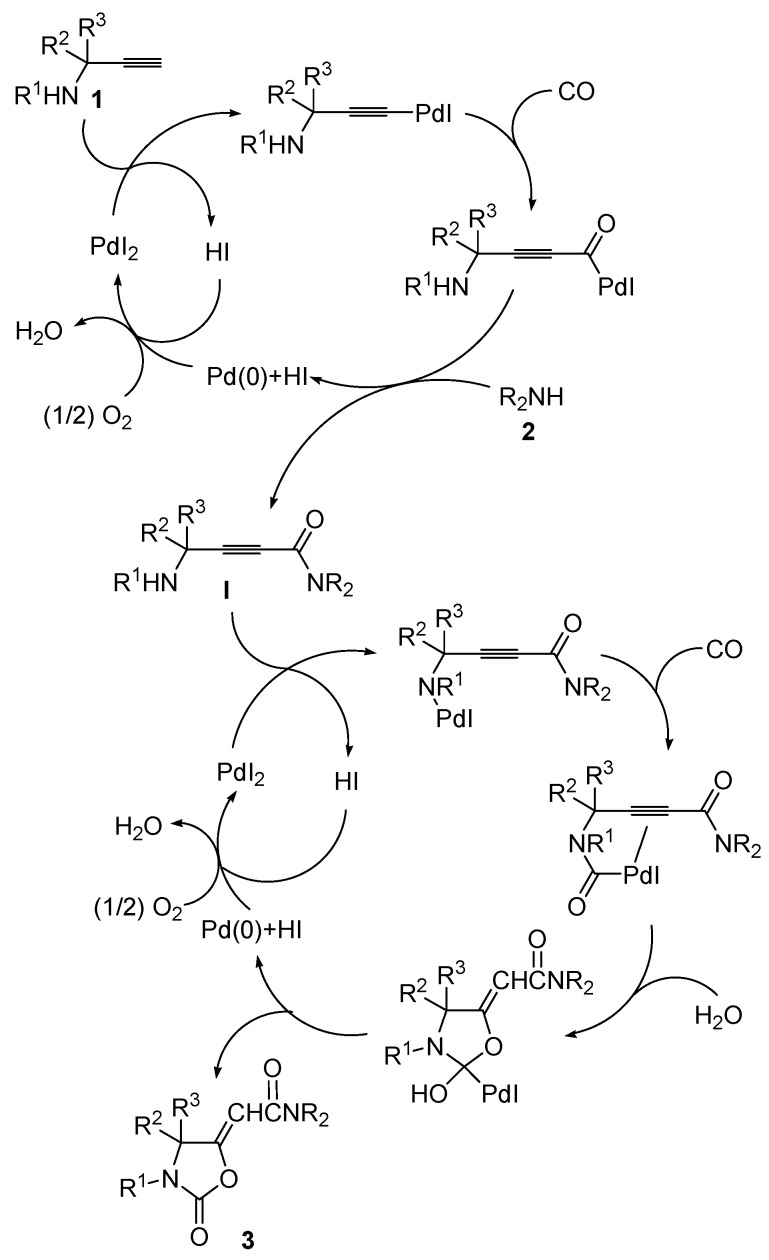
Auto-tandem catalysis leading to oxazolidinones **3** by sequential PdI_2_/KI-catalyzed oxidative monoaminocarbonylation of propargylic amines **1** to give 2-ynamide intermediates **I** followed by PdI_2_/KI-catalyzed and water-promoted oxidative cyclocarbonylation of **I** (anionic iodide ligands are omitted for clarity).

Some years ago, we reported a novel method for the synthesis of 2-(2-oxooxazolidin-5-ylidene)acetamides **3** based on the PdI_2_/KI-catalyzed oxidative carbonylation [12,13,14,15,16,17,18,19] of substituted propargylic amines **1**, carried out in the presence of a secondary amine **2** as external nucleophile, water as a promoter, and molecular oxygen as oxidant [20]. The process, carried out at 100 °C in 1,2-dimethoxyethane (DME) as the solvent under 20 atm (at 25 °C) of a 4:1 mixture of CO/air, led to the formation of a *Z*/*E* mixture of **3** through the concatenation of two catalytic cycles, both catalyzed by PdI_2_/KI, so it represented an example of auto-tandem catalysis. The first process corresponded to the oxidative aminocarbonylation of the triple bond [21] of **1** with **2**, CO, and O_2_, to give 2-ynamide intermediates **I**, while the second process corresponded to the water-promoted oxidative cyclocarbonylation of **I** to give the final products (Scheme 2; anionic iodide ligands are omitted for clarity) [20].

Considering the importance of the class of products obtained, which are known to possess important pharmacological activities [22,23,24,25], and the current attention devoted to the possibility to carry out catalytic processes in ionic liquids (ILs) as safer and more environmentally friendly solvents with respect to classical VOCs [26,27,28,29,30], coupled to the possibility to recycle the catalytic system, we have herein explored the possibility to perform our process in the ionic liquid EmimEtSO_4_. 

Our first experiment was carried out using *N*-benzyl-2-methylbut-3-yn-2-amine (**1a**) as the substrate. This compound was allowed to react with CO, morpholine (**2a**), O_2_, and water in the presence of the catalytic system PdI_2_/KI under the following conditions: PdI_2_/KI/**1a**/**2a**/H_2_O molar ratio = 1:10:50:250:250, T = 100 °C, P(CO) = 16 atm, P(air) = 4 atm, EmimEtSO_4_ as the solvent (0.5 mmol of **1a** per mL of solvent) (Table 1, entry 1, run 1). After 24 h, the reaction crude was extracted several times with diethyl ether and the collected ethereal phases analyzed by TLC, GLC and GC-MS, who revealed the formation of two products whose MS spectra were compatible with the expected isomeric oxazolidinone products **3aa**-*Z* and **3aa**-*E*. The two isomers were isolated by column chromatography and their structure confirmed by IR, ^1^H-NMR and ^13^C-NMR spectroscopies (*Z*/*E* ratio = 3.1:1, total yield = 70%). The yield turned out to be lower using only 2 equiv of **2a** instead of 5 (total yield = 55%). These results confirmed the possibility to carry out the auto-tandem catalysis process leading to oxazolidinones **3** in an ionic liquid (IL) as unconventional solvent. We accordingly verified the possibility to recycle the catalyst/solvent system. Thus, the residue obtained after product extraction with diethyl ether (still containing the catalyst dissolved in the IL), after drying under vacuum (to eliminate the residual diethyl ether), was used again by adding to it fresh propargylamine **1a** and morpholine (**2a**) (1:5 ratio). After stirring at 100 °C for 24 h, **3aa** was obtained again as a 3.2:1 *Z*/*E* mixture in 71% total isolated yield (Table 1, entry 1, run 2), after extraction with diethyl ether. The recycling procedure was then successfully repeated up to six times.

In order to assess the generality of the method, the reaction was then performed using different combinations of propargylic amines **1a**–**e** and secondary amines **2a**–**d**, with the results shown in Table 1, entries 2–9. As can be seen from Table 1, the method could be successfully applied to propargylic amines bearing various alkyl groups α to the triple bond, including simple alkyl groups (such as methyl and ethyl (**1a**, **1b**, and **1d**) and a cyclic chain such as –(CH_2_)_4_, (**1c**)), different groups on nitrogen (such as benzyl (**1a**–**c**) and butyl (**1e**, **1f**)), and different nucleophilic secondary amines, both cyclic (as in the case of morpholine (**2a**), piperidine (**2b**), and pyrrolidine (**2c**)) and acyclic (as in the case of diethylamine (**2d**)). In all cases, good yields of the corresponding 2-(2-oxooxazolidin-5-ylidene)acetamides **3** were obtained (69%–76%), and the PdI_2_/KI/EmimEtSO_4_ system could be conveniently recycled up to six times without loss of activity.

## 3. Experimental Section

### 3.1. General Information

Chemicals were purchased from Sigma-Aldrich Italia (Milano, Italy) and were used as such without further purification. Melting points were taken on a Reichert Thermovar apparatus and are uncorrected. ^1^H-NMR and ^13^C-NMR spectra were recorded at 25 °C in CDCl_3_ solutions with a Bruker DPX Avance 300 spectrometer (Bruker Italia, Milano, Italy) operating at 300 MHz and 75 MHz, respectively, with Me_4_Si as internal standard. IR spectra were taken with a JASCO FT-IR 4200 spectrometer (Jasco Europe s.r.l., Cremella, Lecco, Italy). Mass spectra were obtained using a Shimadzu QP-2010 GC-MS apparatus (Shimadzu Italia, Milano, Italy) at 70 eV ionization voltage. Microanalyses were carried out with a Thermo-Fischer Elemental Analyzer Flash 2000 (Fischer Scientific Italia, Rodani, Milano, Italy). All reactions were analyzed by TLC (Merck Italy, Vimodrone, Milano, Italy) on silica gel 60 F254 (Merck Italy) or on neutral alumina (Merck Italy) and by GLC using a Shimadzu GC-2010 gas chromatograph (Shimadzu Italia) and capillary columns with polymethylsilicone + 5% polyphenylsilicone as the stationary phase (HP-5). Column chromatography was performed on neutral alumina 90 (Merck Italy, 70–230 mesh). Evaporation refers to the removal of solvent under reduced pressure.

### 3.2. Preparation of Substrates

Starting propargylic amines **1a**–**e** were prepared and characterized as already described [20].

### 3.3. Preparation of EmimEtSO_4_

Ionic liquid 1-ethyl-3-methylimidazolium ethyl sulfate (EmimEtSO_4_) was prepared as previously described [31].

### 3.4. General Procedure for the PdI_2_/KI-Catalyzed Oxidative Carbonylation of Propargylic Amines (***1***) with CO, O_2_, and Secondary Amines (***2***) in EmimEtSO_4_ and Recycling Experiments

A 35 mL stainless steel autoclave was charged with PdI_2_ (8.3 mg, 0.023 mmol), KI (38.3 mg, 0.23 mmol), the starting propargylic amine **1** (**1a**, 199.0 mg; **1b**, 215.0 mg; **1c**, 245.0 mg; **1d**, 176.0 mg; **1e**, 206.0 mg; 1.15 mmol) and the amine **2** (**2a**, 501.0 mg; **2b**, 490.0 mg; **2c**, 409.0 mg; **2d**, 420.6 mg; 5.75 mmol) in EmimEtSO_4_ (2.3 mL). Water (103.5 μL, 5.75 mmol) was then added and the autoclave was sealed, and pressurized at 20 atm (16 atm CO and 4 atm Air). After stirring at 100 °C for 24 h, the autoclave was cooled and degassed. The mixture was then extracted with Et_2_O (6 × 4 mL), and the residue (still containing the catalyst and water dissolved in the ionic liquid) was used as such for the next recycle (see below). The collected ethereal phases were concentrated and the products purified by column chromatography on neutral alumina using as the eluent hexane:AcOEt from 95:5 to 6:4 (the *E* isomers were eluted first in all cases). (**3aa**-*E*, 64.8 mg, 17%; **3aa**-*Z*, 201.4 mg, 53%; **3ab**-*E*, 68.0 mg, 18%; **3ab**-*Z*, 211.5 mg, 56%; **3ac**-*E*, 54.5 mg, 15%; **3ac**-*Z*, 216.7 mg, 60%; **3ad**-*E*, 61.9 mg, 17%; **3ad**-*Z*, 192.8 mg, 53%; **3ba**-*E*, 75.2 mg, 19%; **3ba**-*Z*, 221.8 mg, 56%; **3ca**-*E*, 42.6 mg, 10%; **3ca**-*Z*, 259.9, 61% mg; **3da***-E*, 39.3 mg, 11%; **3da**-*Z*, 207.0 mg, 58%; **3dd**-*E***,** 34.1 mg, 10%; **3ea**-*E*, 50.3 mg, 13%; **3ea**-*Z*, 216.7 mg, 56%). *Note:* Product **3dd**-*Z* could not be isolated at the pure state by column chromatography, and the GLC analysis evidenced a purity of ca. 60%. The GLC-MS analysis was compatible with the proposed structure and ^1^H-NMR spectrum of the crude product evidenced a peak at 5.19, compatible with a Z stereochemistry. For testing recycling of the catalyst, after removal of Et_2_O under vacuum, the residue obtained as described above, still containing the catalyst dissolved in the ionic liquid, was transferred into the autoclave. The starting material **1** (1.15 mmol), the amine **2** (5.75 mmol) and H_2_O (103.5 μL, 5.75 mmol) was added, and then the same procedure described above was followed.

### 3.5. Characterization of Products

Oxazolidinones **3aa**-*Z*, **3aa**-*E*, **3ab**-*Z*, **3ab**-*E*, **3ad**-*Z*, **3ad**-*E*, **3ba**-*Z*, **3ba**-*E*, **3ca**-*Z*, **3ca**-*E*, **3da**-*Z*, **3da**-*E*, **3ea**-*Z*, **3ea**-*E*, were characterized by comparison with the characterization data already reported by us [20]. All the other products were fully characterized by MS spectrometry, IR, ^1^H-NMR and ^13^C-NMR spectroscopies, and elemental analysis, as reported below. Copies of Copies of ^1^H-NMR and ^13^C-NMR spectra for all products are given in the Supplementary Materials.

*(E)-3-Benzyl-4,4-dimethyl-5-(2-oxo-2-pyrrolidin-1-ylethylidene)-oxazolidin-2-one (***3ac***-E).* White solid, m.p. = 124–125 °C. IR (KBr): ν = 1779 (s), 1664 (m), 1611 (m), 1404 (m), 1346 (w), 1194 (w), 709 (w) cm^−1^; ^1^H-NMR (CDCl_3_): δ = 7.38–7.26 (m, 5 H, aromatic), 5.83 (s, 1 H, =CH), 4.47 (s, 2H, C*H*_2_Ph), 3.51–3.40 (m, 4H, pyrrolidine ring), 2.02–1.82 (m, 4H, pyrrolidine ring), 1.64 (s, 6H, 2Me); ^13^C-NMR (CDCl_3_): δ = 165.4, 162.9, 153.6, 137.3, 128.7, 127.8, 96.8, 64.4, 47.2, 45.7, 43.8, 26.2, 24.4, 23.9; GC-MS *m/z* = 314 (11) [M^+^], 299 (5), 286 (2), 255 (1), 244 (2), 223 (13), 216 (2), 181 (2), 166 (4), 146 (2), 140 (8), 132 (2), 112 (2), 98 (9), 91 (100); anal. calcd for C_18_H_22_N_2_O_3_ (314.38): C, 68.77; H, 7.05; N, 8.91; found C, 68.75; H, 7.04; N, 8.89.

*(Z)-3-Benzyl-4,4-dimethyl-5-(2-oxo-2-pyrrolidin-1-ylethylidene)-oxazolidin-2-one (***3ac**-*Z).* Yellow solid, m.p. = 105–106 °C. IR (KBr): ν = 1780 (s), 1685 (s), 1616 (m), 1438 (m), 1401 (m), 1225 (w), 1036 (m), 754 (w) cm^−^^1^; ^1^H-NMR (CDCl_3_): δ = 7.48–7.22 (m, 5H, aromatic), 5.21 (s, 1H, =CH), 4.48 (s, 2H, C*H*_2_Ph), 3.59–3.43 (m, 4H, pyrrolidine ring), 2.03–1.84 (m, 4H, pyrrolidine ring), 1.35 (s, 6H, 2Me); ^13^C-NMR (CDCl_3_): δ = 162.7, 160.5, 153.8, 137.1, 128.8, 128.0, 127.8, 94.6, 62.4, 47.1, 45.7, 44.2, 27.5, 25.9, 24.4; GC-MS *m/z* = 314 (8) [M^+^], 299 (4), 286 (1), 255 (1), 243 (3), 223 (11), 216 (1), 181 (2), 166 (2), 147 (4), 140 (6), 132 (1), 112 (2), 98 (7), 91 (100); anal. calcd for C_18_H_22_N_2_O_3_ (314.38): C, 68.77; H, 7.05; N, 8.91; found C, 68.78; H, 7.03; N, 8.92.

*(E)-2-(3-Butyl-4-ethyl-4-methyl-2-oxooxazolidin-5-ylidene)-N,N-diethylacetamide (***3dd***-E).* Yellow oil. IR (film): ν = 1786 (s), 1687 (m), 1618 (m), 1401 (w), 1052 (m), 1082 (m) cm^−1^; ^1^H-NMR (CDCl_3_): δ = 5.92 (s, 1H, =C*H*), 3.46–3.30 (m, 4H, 2NCH_2_), 3.29–3.16 (m, 1H, C*H*H), 3.06–2.93 (m, 1H, C*H*H), 2.76–2.60 (m, 1H, C*H*H), 1.70–1.53 (m, 3H, CH_2_ + CH*H*), 1.42–1.30 (m, 2H, CH_2_), 1.66 (s, 3H, Me), 1.19 (t, *J* = 7.2, 3H, Me), 1.14 (t, *J* = 7.1, 3H, Me), 0.95 (t, *J* = 7.4, 3H, Me), 0.79 (t, *J* = 7.4, 3H, Me); ^13^C-NMR (CDCl_3_): δ = 164.1, 163.5, 153.8, 96.7, 68.0, 43.0, 40.5, 40.0, 31.1, 28.5, 23.7, 20.3, 14.5, 13.7, 13.1, 8.2; GC-MS *m/z* = 296 (25) [M^+^], 281 (15), 267 (100), 224 (8), 180 (15), 168 (98), 140 (15), 124 (19), 112 (9), 100 (54), 72 (71); anal. calcd for C_16_H_28_N_2_O_3_ (296.41): C, 64.83; H, 9.52; N, 9.45; found C, 64.80; H, 9.54; N, 9.42.

*(Z)-2-(3-Butyl-4-ethyl-4-methyl-2-oxooxazolidin-5-ylidene)-N,N-diethylacetamide (***3dd**-*Z).* Yellow oil, purity: ca. 60% (by GLC). GC-MS *m/z* =296 (26) [M^+^], 281 (11), 267 (100), 224 (13), 197 (8), 180 (26), 168 (74), 138 (13), 124 (33), 112 (14), 100 (37), 72 (89).

## 4. Conclusions

In conclusion, we have shown that it is possible to successfully perform the PdI_2_/KI-catalyzed auto-tandem catalysis oxidative carbonylative process leading to oxazolidone derivatives **3** in an ionic liquid, such as EmimEtSO_4_, as unconventional solvent, and that the catalyst/solvent system can be easily recycled several times without loss of activity.

## Figures and Tables

**Table 1:**
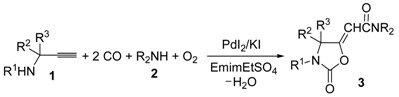
Synthesis of 2-(2-oxooxazolidin-5-ylidene)acetamides (**3**) by PdI_2_/KI-catalyzed oxidative carbonylation of propargylic amines (**1**) with CO, O_2_, and secondary amines (**2**) in EmimEtSO_4_ and recycling experiments ^a^.

Entry	1	2	3	Yield of 3 (%) ^b^ (*Z*/*E* ratio) ^c^						
				Run 1	Run 2	Run 3	Run 4	Run 5	Run 6	Run 7 ^d^
1	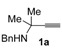	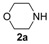	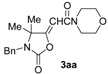	70 (3.1)	71 (3.2)	74 (2.9)	74 (3.4)	71 (3.2)	70 (3.4)	70 (3.2)
2	**1a**	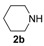	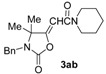	74 (3.1)	75 (3.4)	73 (3.2)	75 (3.0)	74 (3.3)	73 (3.2)	74 (3.3)
3	**1a**	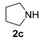	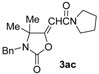	75 (4.0)	75 (3.9)	74 (3.9)	76 (4.0)	76 (3.9)	75 (3.7)	75 (3.8)
4	**1a**	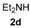	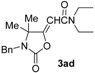	70 (3.1)	71 (2.9)	71 (3.1)	70 (3.4)	70 (3.4)	69 (3.0)	71 (3.1)
5	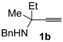	**2a**	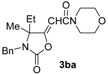	75 (2.9)	75 (3.4)	74 (3.3)	75 (3.0)	76 (3.1)	74 (3.1)	74 (3.2)
6	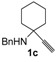	**2a**	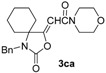	71 (6.1)	74 (7.2)	72 (6.9)	74 (6.4)	73 (7.1)	75 (7.3)	74 (7.3)
7	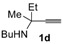	**2a**	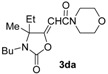	69 (5.3)	70 (5.4)	70 (6.0)	71 (5.5)	72 (5.0)	69 (5.9)	72 (5.6)
8	**1d**	**2d**	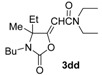	74 (6.4) ^e^	76 (5.9) ^e^	76 (6.0) ^e^	75 (6.3) ^e^	74 (6.2) ^e^	75 (6.1) ^e^	74 (6.0) ^e^
9	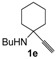	**2a**	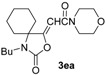	69 (4.3)	72 (4.5)	71 (4.5)	71 (4.9)	72 (4.5)	72 (4.5)	70 (4.8)

^a^ All reactions were carried out in the presence of PdI_2_, KI, and H_2_O at 100 °C under 20 atm (at 25 °C) of a 4:1 mixture CO/air, in EmimEtSO_4_ for 24 h with a substrate concentration of 0.5 mmol of **1** per mL of ionic liquid. The H_2_O:**2**:**1**:KI:PdI_2_ molar ratio was 250:250:50:10:1. Conversion of **1** was quantitative in all cases. ^b^ Isolated yield (*Z*+*E*) based on starting **1**. ^c^ Determined by isolation of the pure diastereoisomers. ^d^ Run 1 corresponds to the 1st experiment, the next runs to recycles. See text for details. ^e^ Determined by GLC. The *E* isomer could be isolated at the pure state after column chromatography, while the Z isomer was isolated with a purity of ca. 60% (by GLC). See the Experimental Section for details.

## Supplementary Materials

Supplementary materials can be accessed at: http://www.mdpi.com/1420-3049/21/7/897/s1.

## Abbreviations

The following abbreviations are used in this manuscript:

DME	1,2-dimethoxyethane
EmimEtSO_4_	1-ethyl-3-methylimidazolium ethyl sulfate
Et_2_O	diethyl ether
GC	gas chromatograph
GC-MS	gas chromatograph/mass spectrometer
GLC	gas-liquid chromatography
GLC-MS	gas-liquid chromatography/mass spectrometry
ILs	Ionic Liquids
TLC	thin layer chromatography
MS	mass
NMR	nuclear magnetic resonance
VOCs	Volatile Organic Compounds
