# Supplementary material for: Auto-Tandem Catalysis in Ionic Liquids: Synthesis of 2-Oxazolidinones by Palladium-Catalyzed Oxidative Carbonylation of Propargylic Amines in EmimEtSO4
Source: Molecules. 2016 Jul 8;21(7):897. doi: 10.3390/molecules21070897 (PMC6273147; doi:10.3390/molecules21070897)
Supplement: Supplementary file 1 [file molecules-21-00897-s001.pdf]

Supplementary Materials: Auto-Tandem Catalysis in Ionic Liquids: Synthesis of 2-Oxazolidinones by Palladium-Catalyzed Oxidative Carbonylation of Propargylic Amines in EmimEtSO4

Raffaella Mancuso, Asif Maner, Ida Ziccarelli, Christian Pomelli, Cinzia Chiappe, Nicola Della Ca', Lucia Veltri, and Bartolo Gabriele

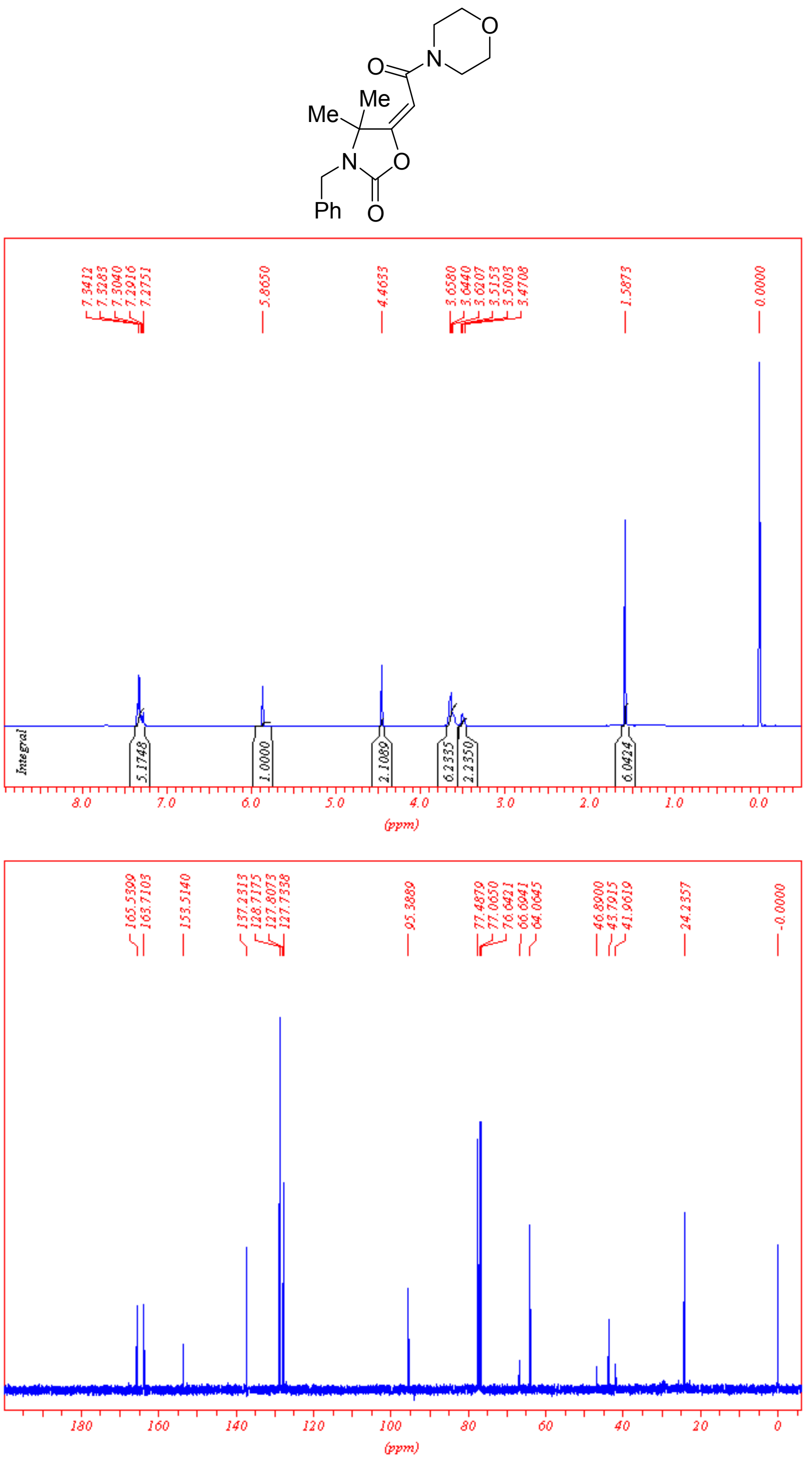

Figure S1. (*E*)-3-Benzyl-4,4-dimethyl-5-(2-morpholin-4-yl-2-oxoethylidene)oxazolidin-2-one (**3aa**-*E*). Top: ^{1}H-NMR ($CDCl_3$); bottom: ^{13}C-NMR ($CDCl_3$).

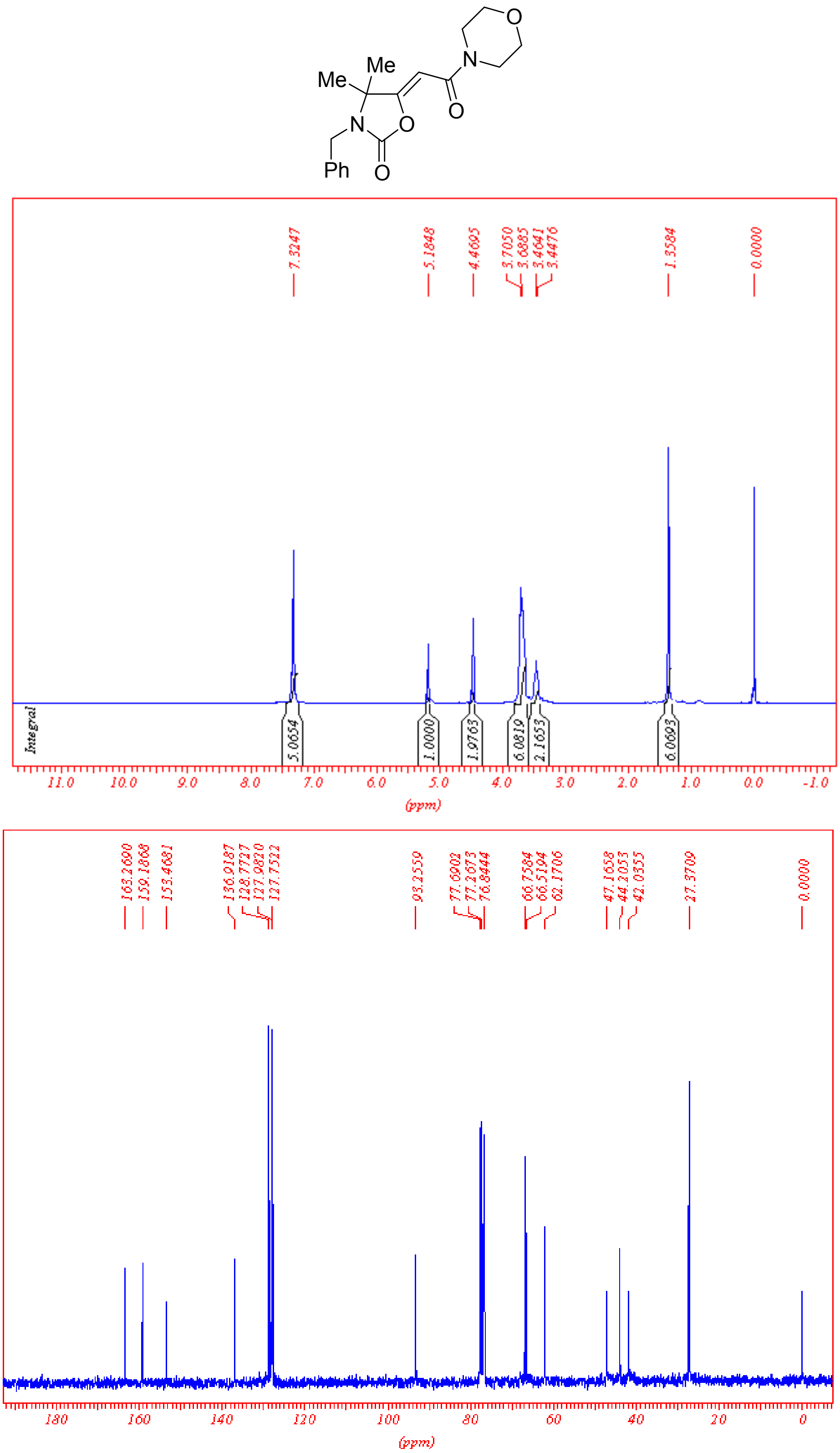

Figure S2. (*Z*)-3-Benzyl-4,4-dimethyl-5-(2-morpholin-4-yl-2-oxoethylidene)oxazolidin-2-one (**3aa**-*Z*).

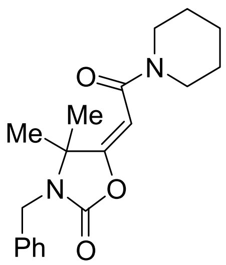

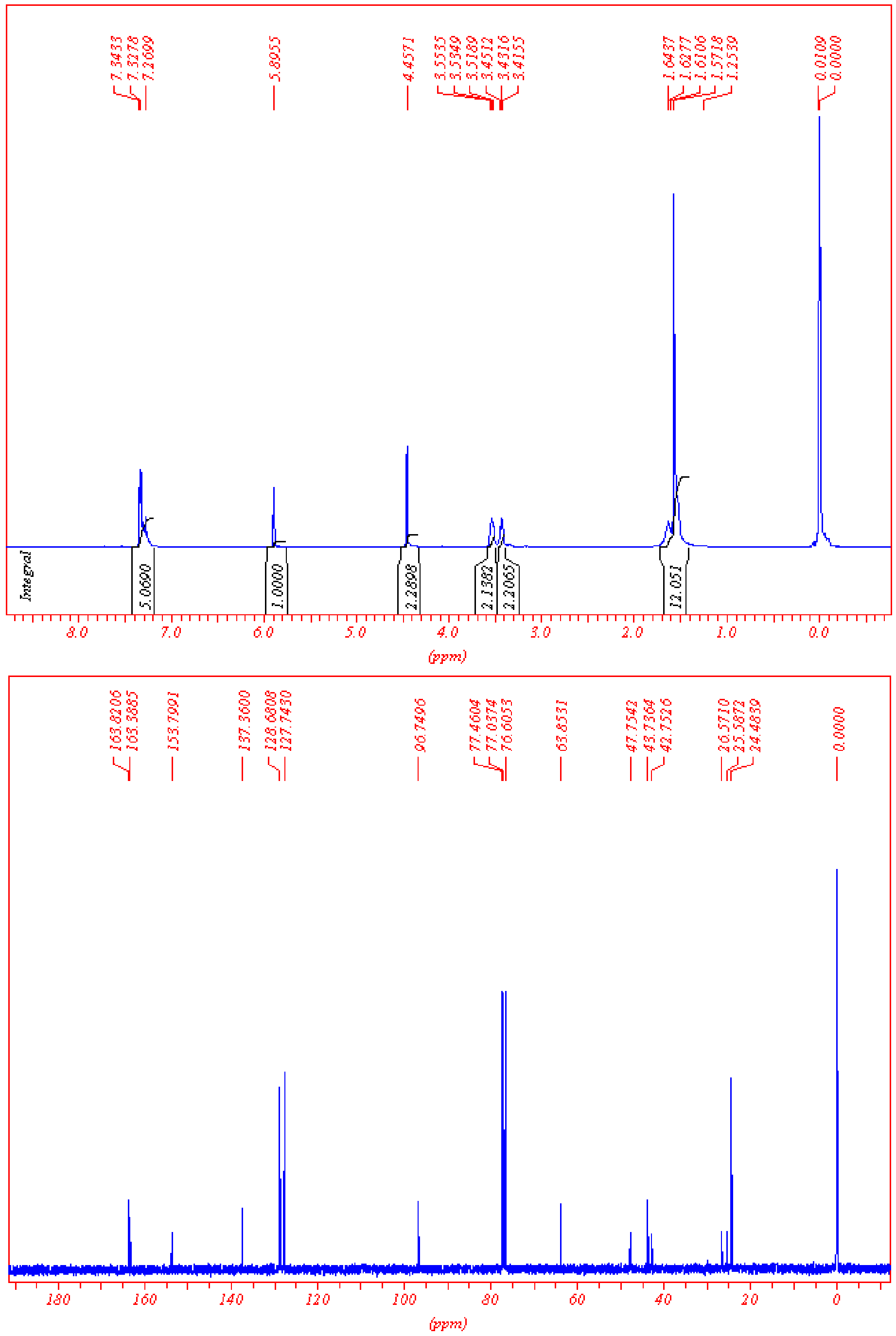

Figure S3. (*E*)-3-Benzyl-4,4-dimethyl-5-(2-oxo-2-piperidin-1-ylethylidene)oxazolidin-2-one (**3ab**-*E*).

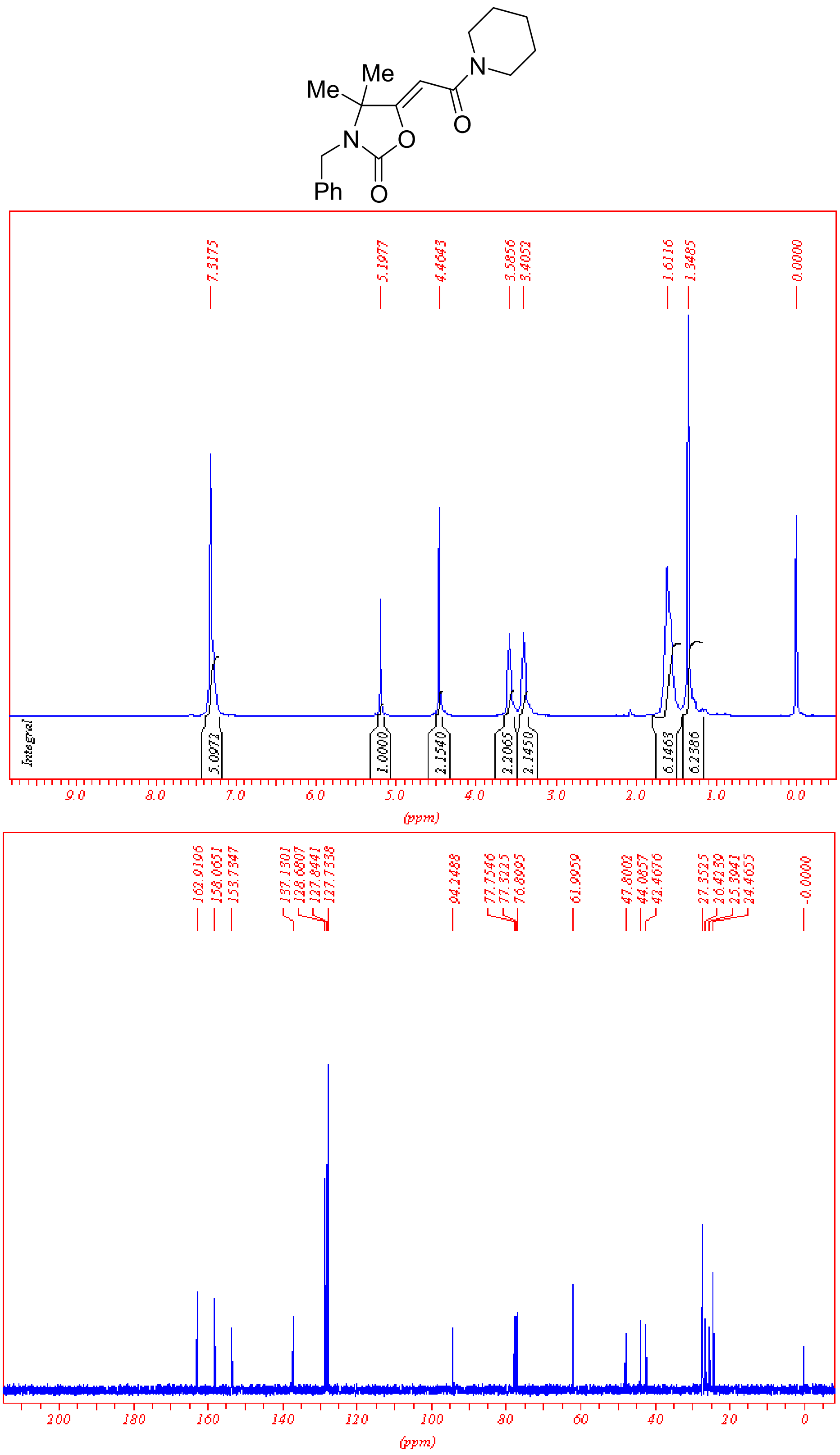

Figure S4. (*Z*)-3-Benzyl-4,4-dimethyl-5-(2-oxo-2-piperidin-1-ylethylidene)oxazolidin-2-one (**3ab**-*Z*).

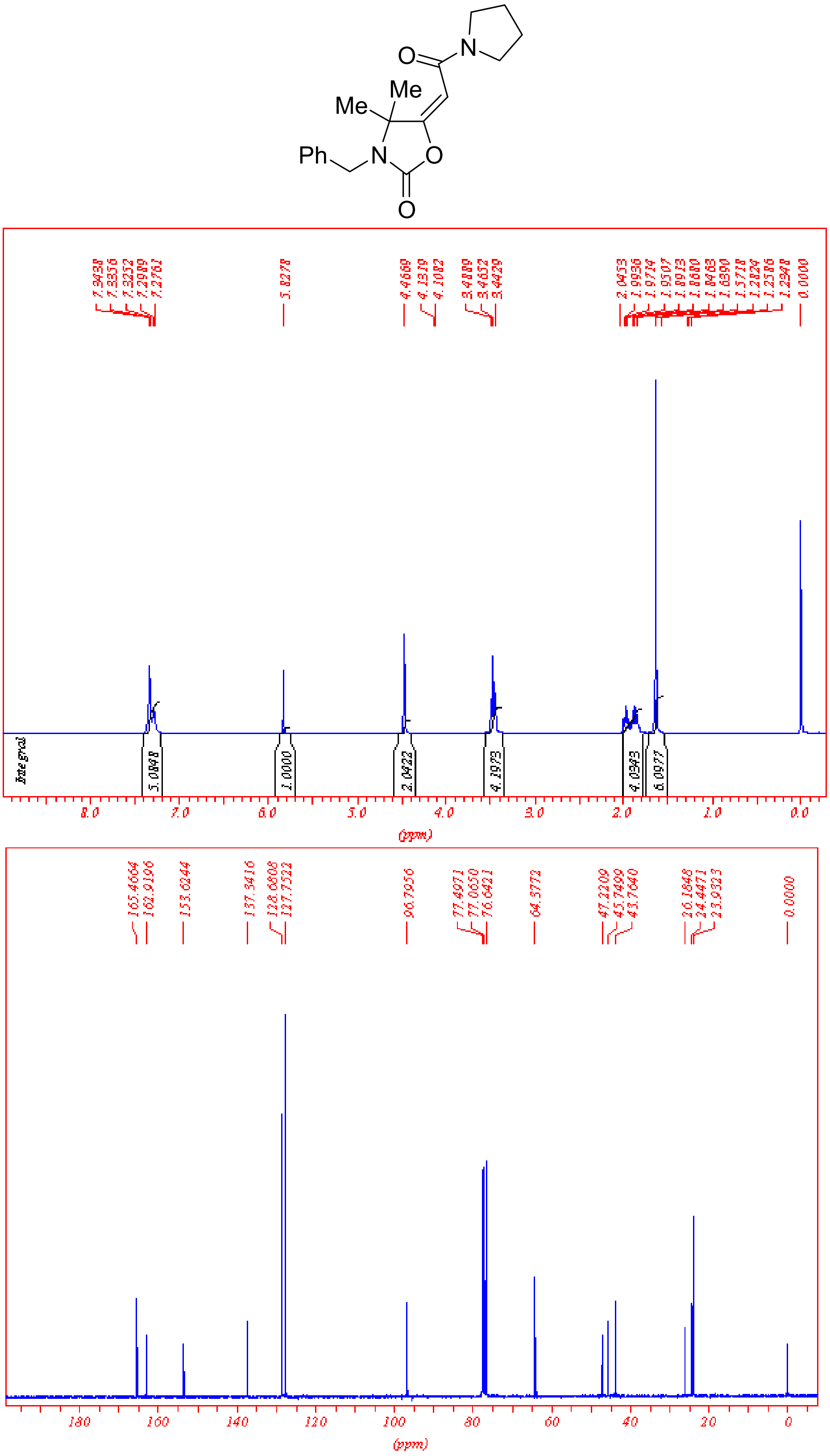

Figure S5 (*E*)-3-Benzyl-4,4-dimethyl-5-(2-oxo-2-pyrrolidin-1-ylethylidene)oxazolidin-2-one (**3ac**-*E*).

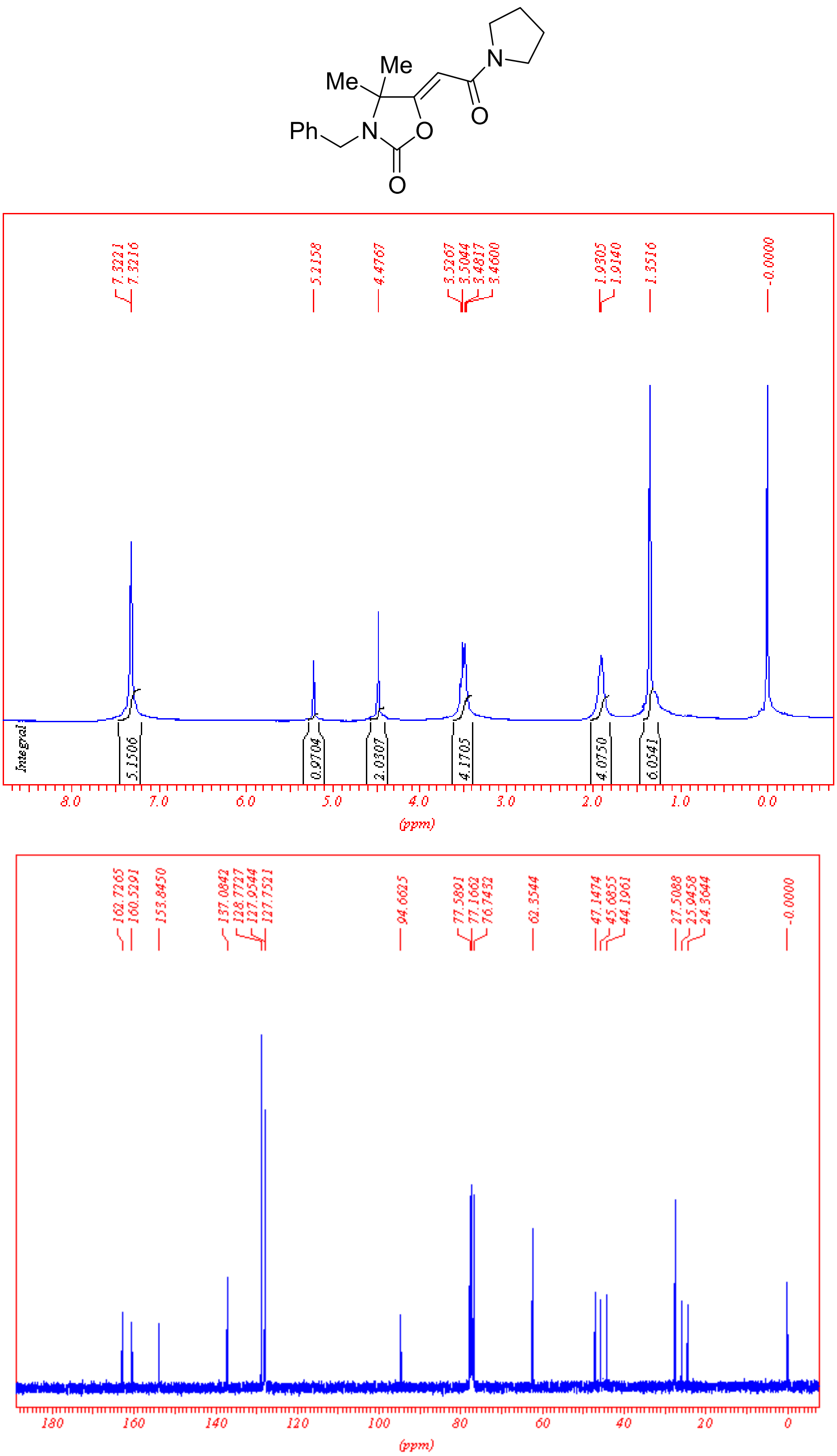

Figure S6. (*Z*)-3-Benzyl-4,4-dimethyl-5-(2-oxo-2-pyrrolidin-1-ylethylidene)oxazolidin-2-one (**3ac**-*Z*).

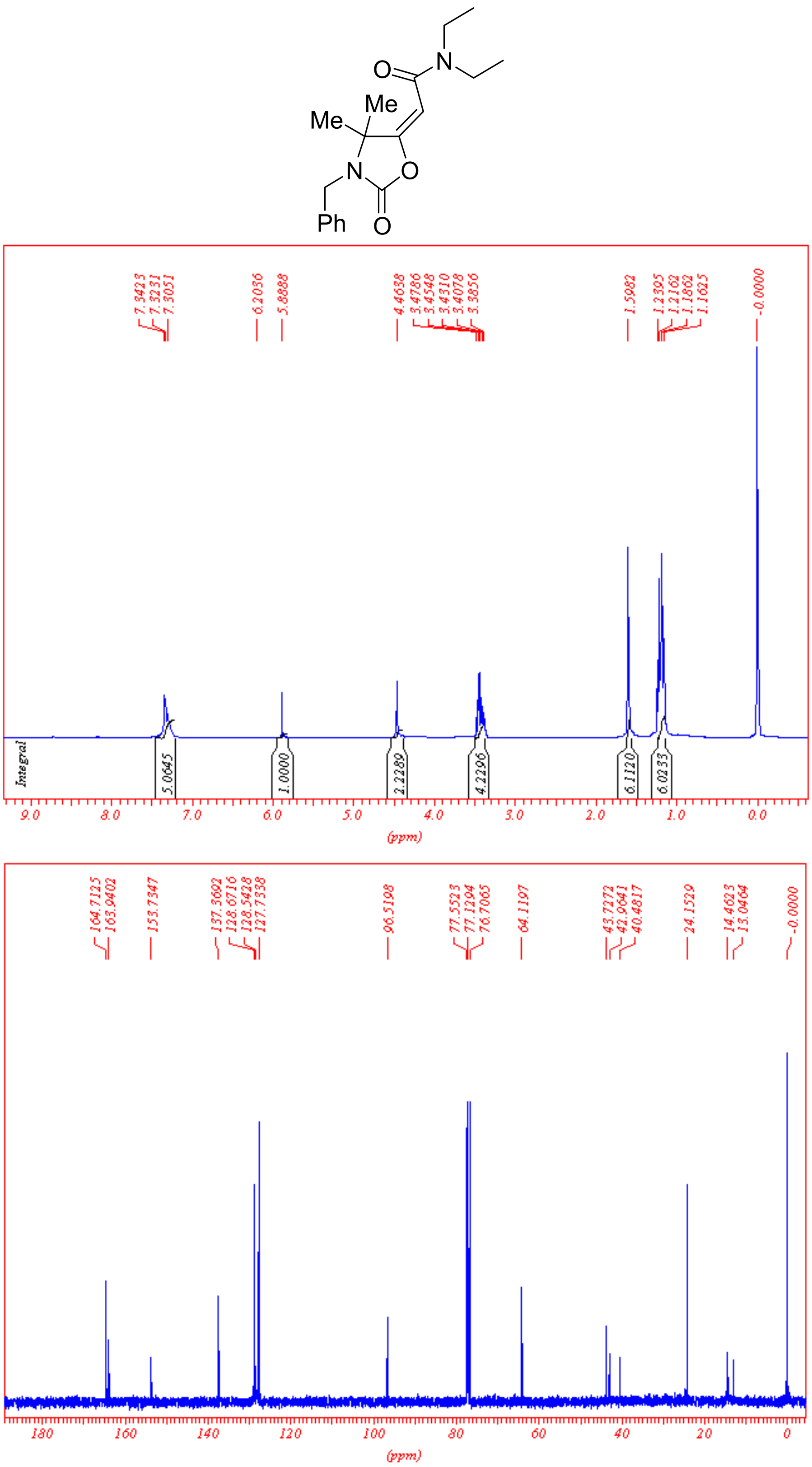

Figure S7. (*E*)-2-(3-Benzyl-4,4-dimethyl-2-oxo-oxazolidin-5-ylidene)-*N*,*N*-diethylacetamide (**3ad**-*E*).

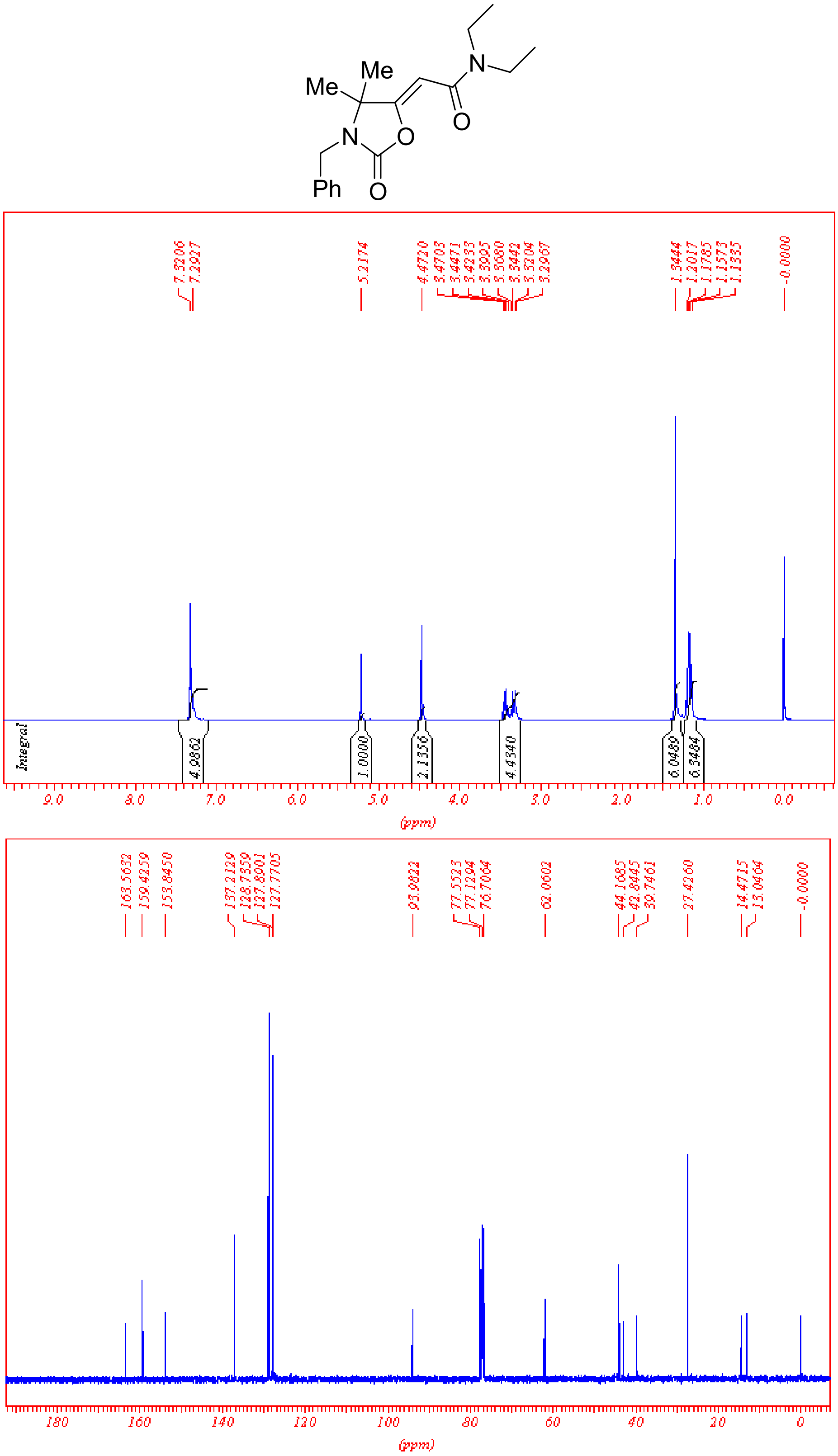

Figure S8. (*Z*)-2-(3-Benzyl-4,4-dimethyl-2-oxo-oxazolidin-5-ylidene)-*N*,*N*-diethylacetamide (**3ad**-*Z*).

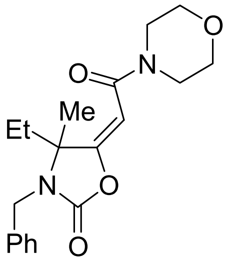

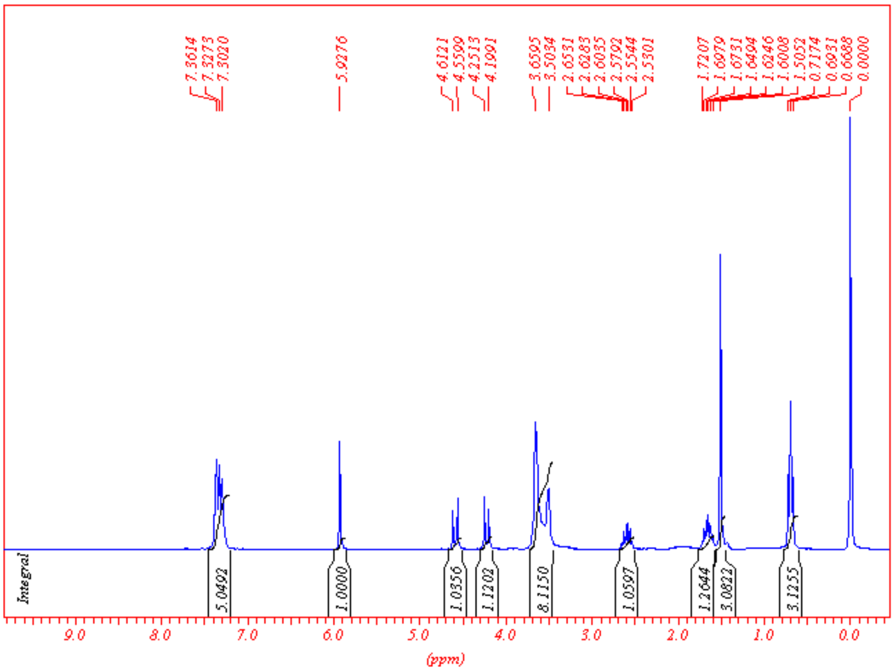

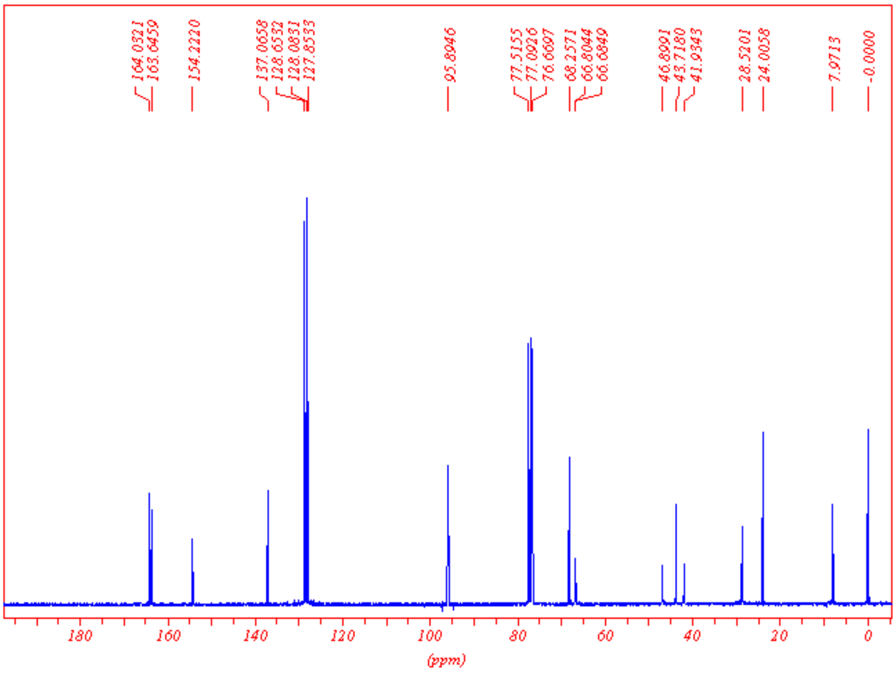

Figure S9. (*E*)-3-Benzyl-4-ethyl-4-methyl-5-(2-morpholin-4-yl-2-oxoethylidene)oxazolidin-2-on*e* (**3ba**-*E*).

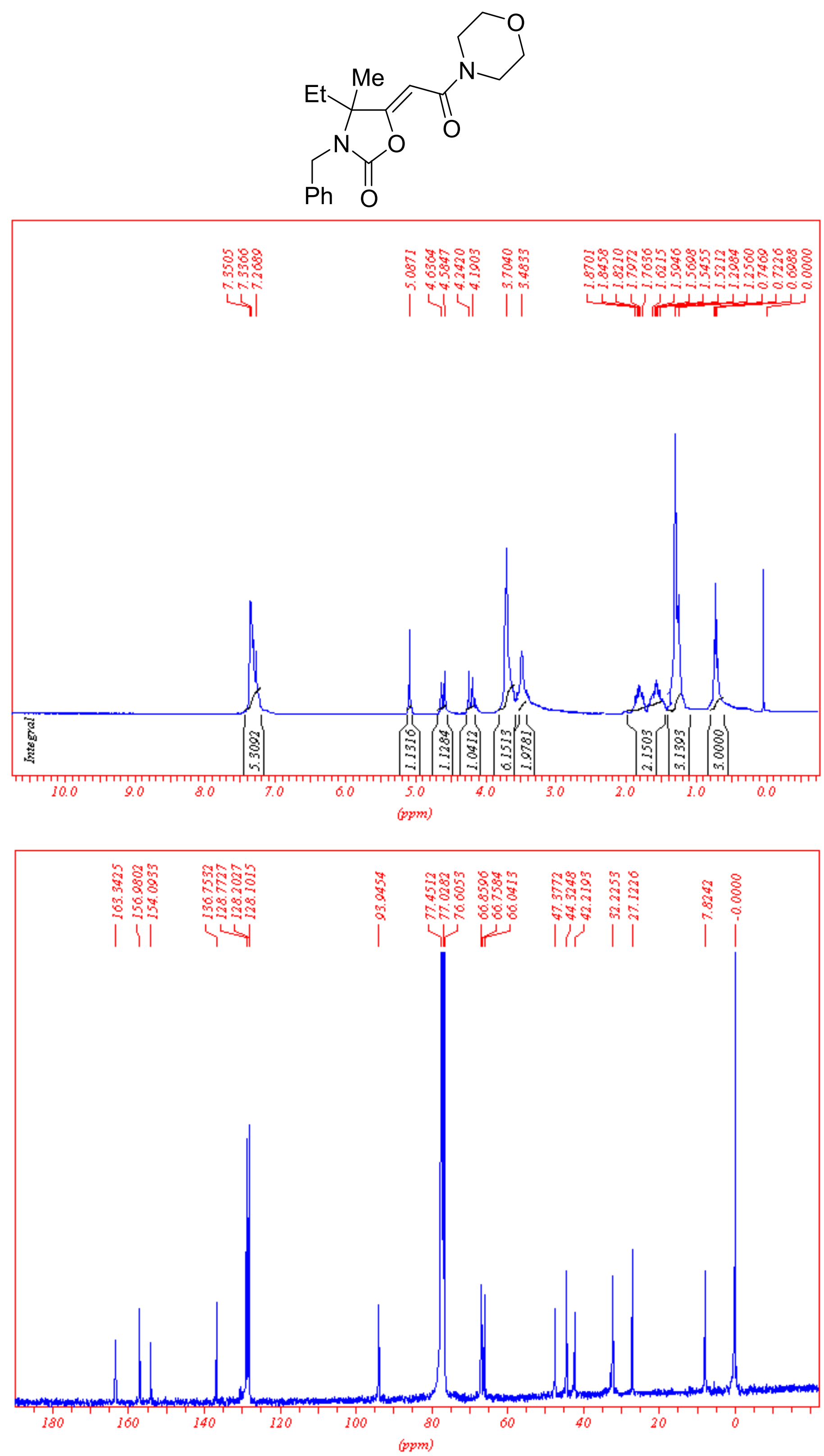

Figure S10. (*Z*)-3-Benzyl-4-ethyl-4-methyl-5-(2-morpholin-4-yl-2-oxoethylidene)oxazolidin-2-one (**3ba**-*Z*).

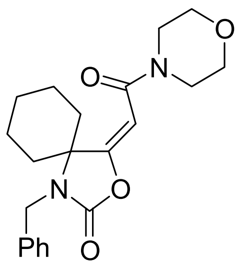

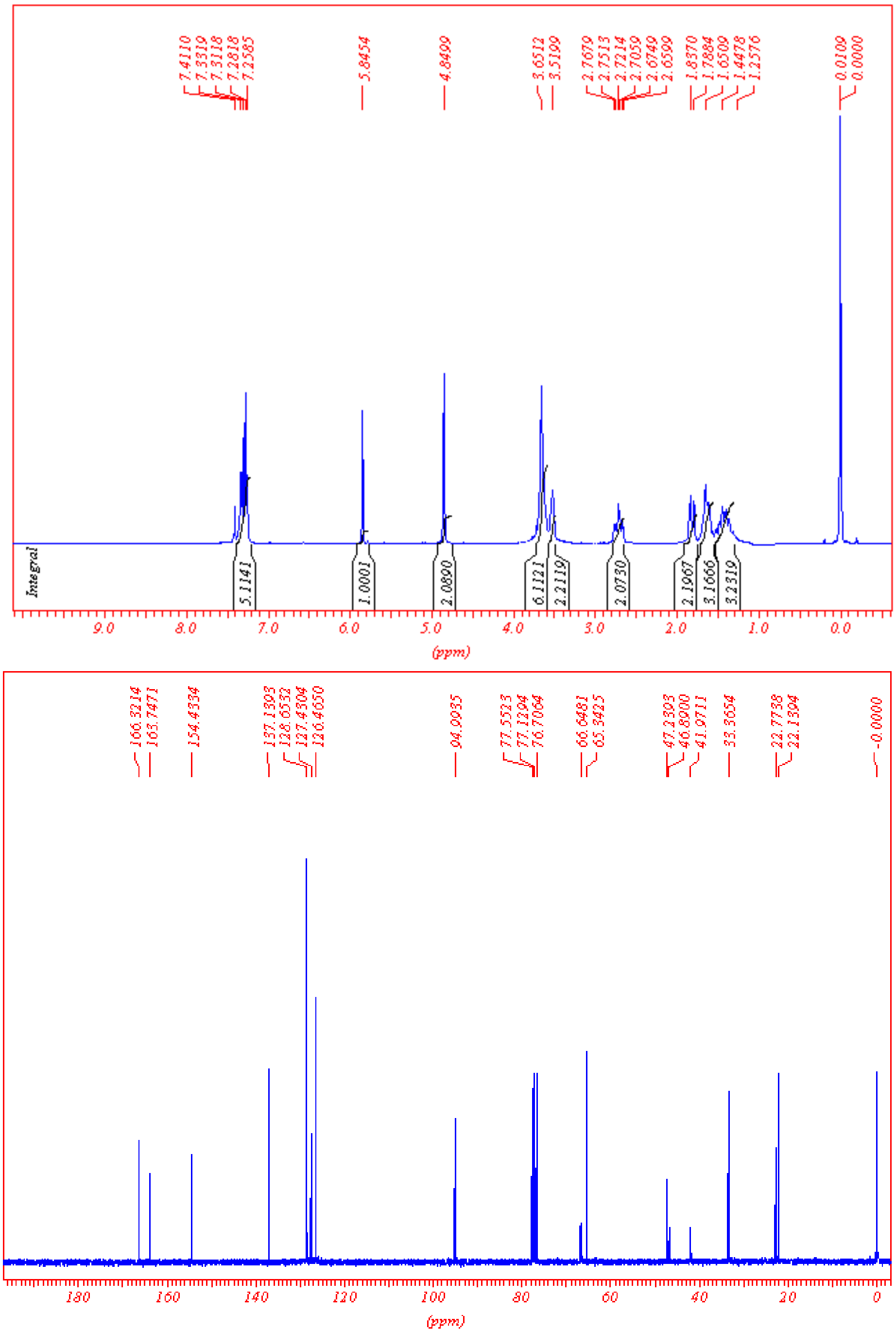

Figure S11. (*E*)-1-Benzyl-4-(2-morpholin-4-yl-2-oxoethylidene)-3-oxa-1-azaspiro[4.5]decan-2-one (**3ca**-*E*).

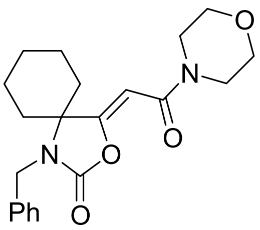

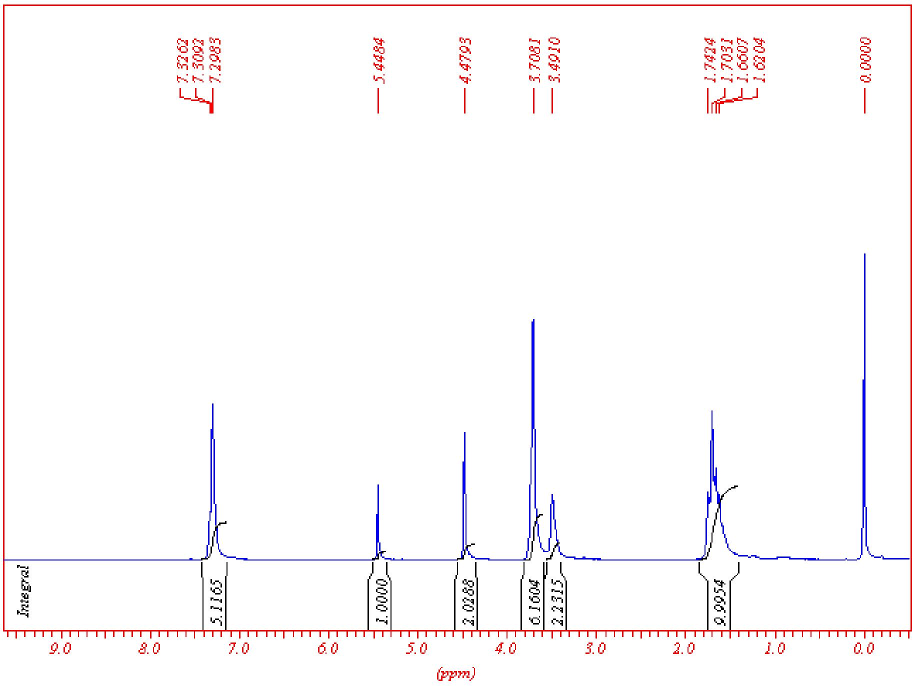

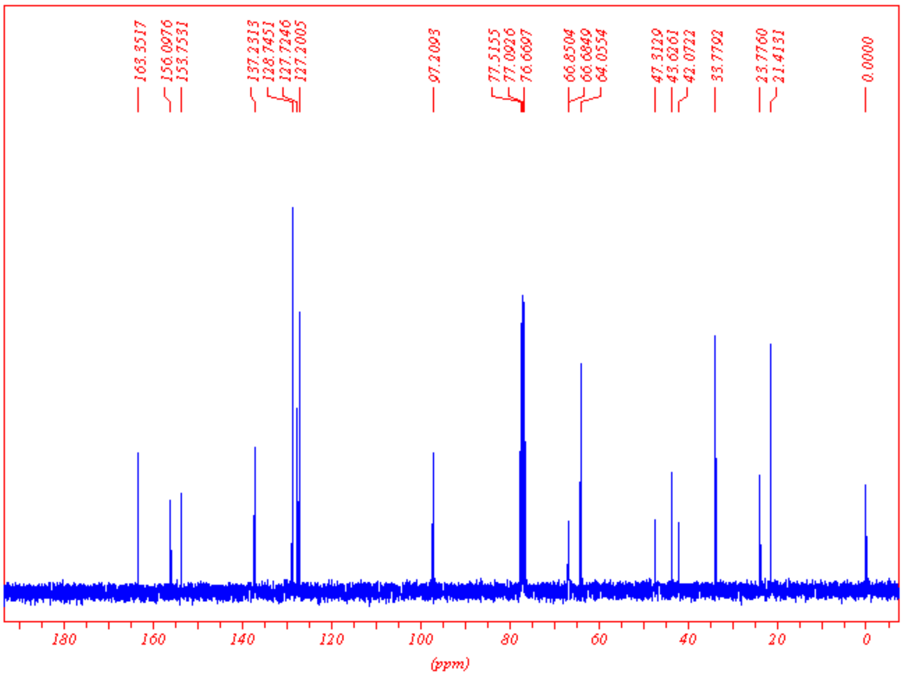

Figure S12. (*Z*)-1-Benzyl-4-(2-morpholin-4-yl-2-oxoethylidene)-3-oxa-1-azaspiro[4.5]decan-2-one (**3ca**-*Z*).

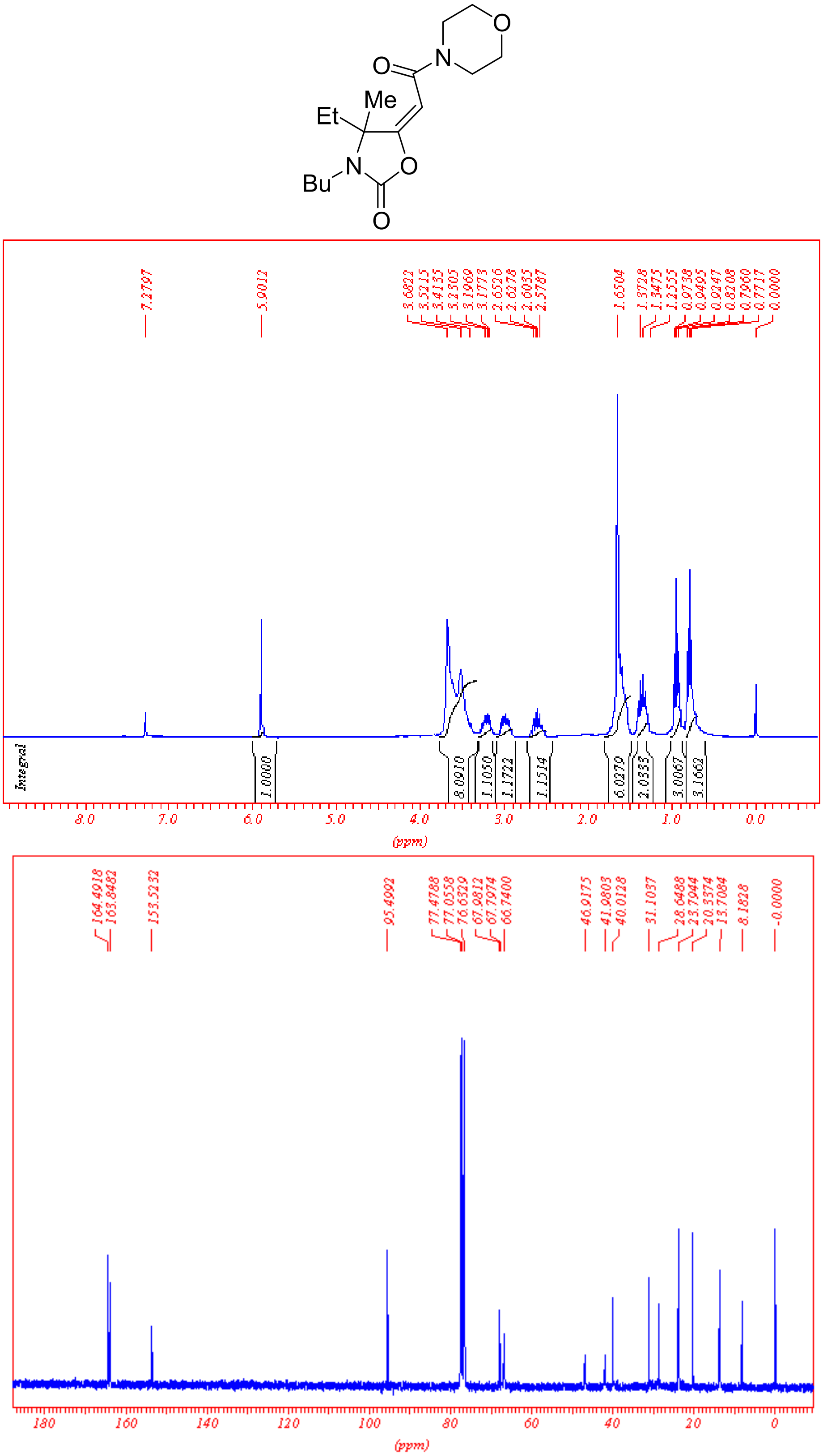

Figure S13. (*E*)-3-Butyl-4-ethyl-4-methyl-5-(2-morpholin-4-yl-2-oxoethylidene)oxazolidin-2-one (**3da**-*E*).

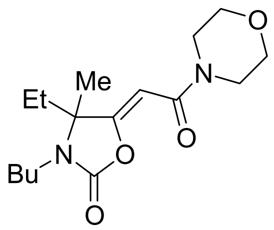

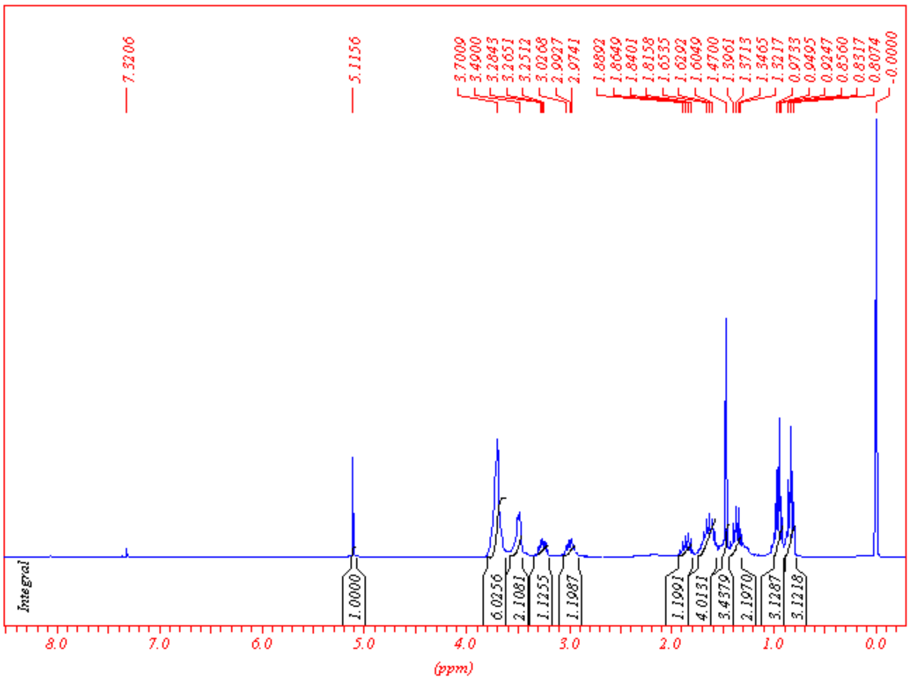

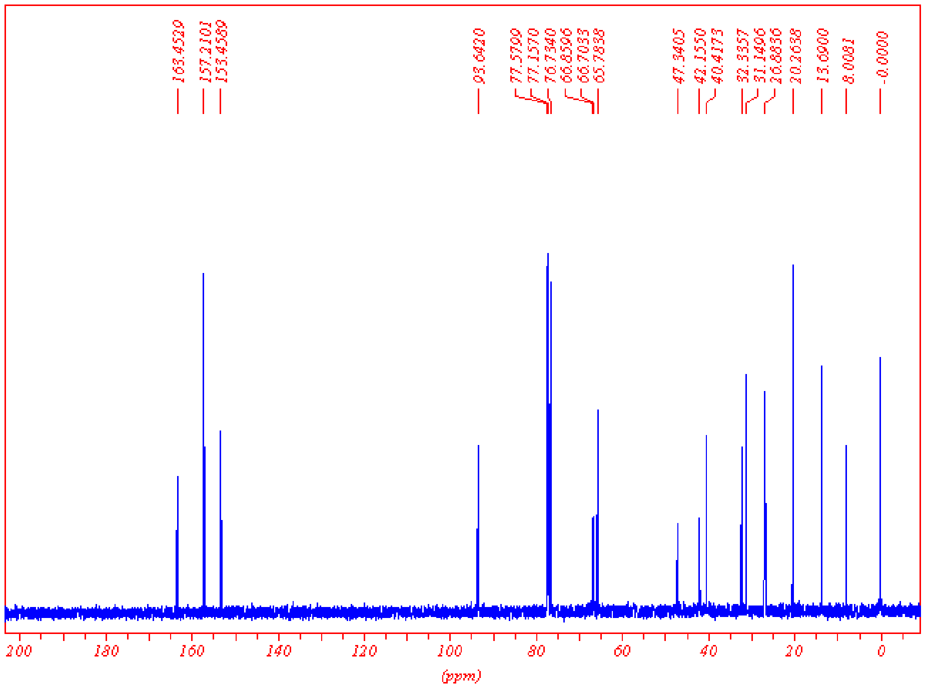

Figure S14. (*Z*)-3-Butyl-4-ethyl-4-methyl-5-(2-morpholin-4-yl-2-oxoethylidene)oxazolidin-2-one (**3da**-*Z*).

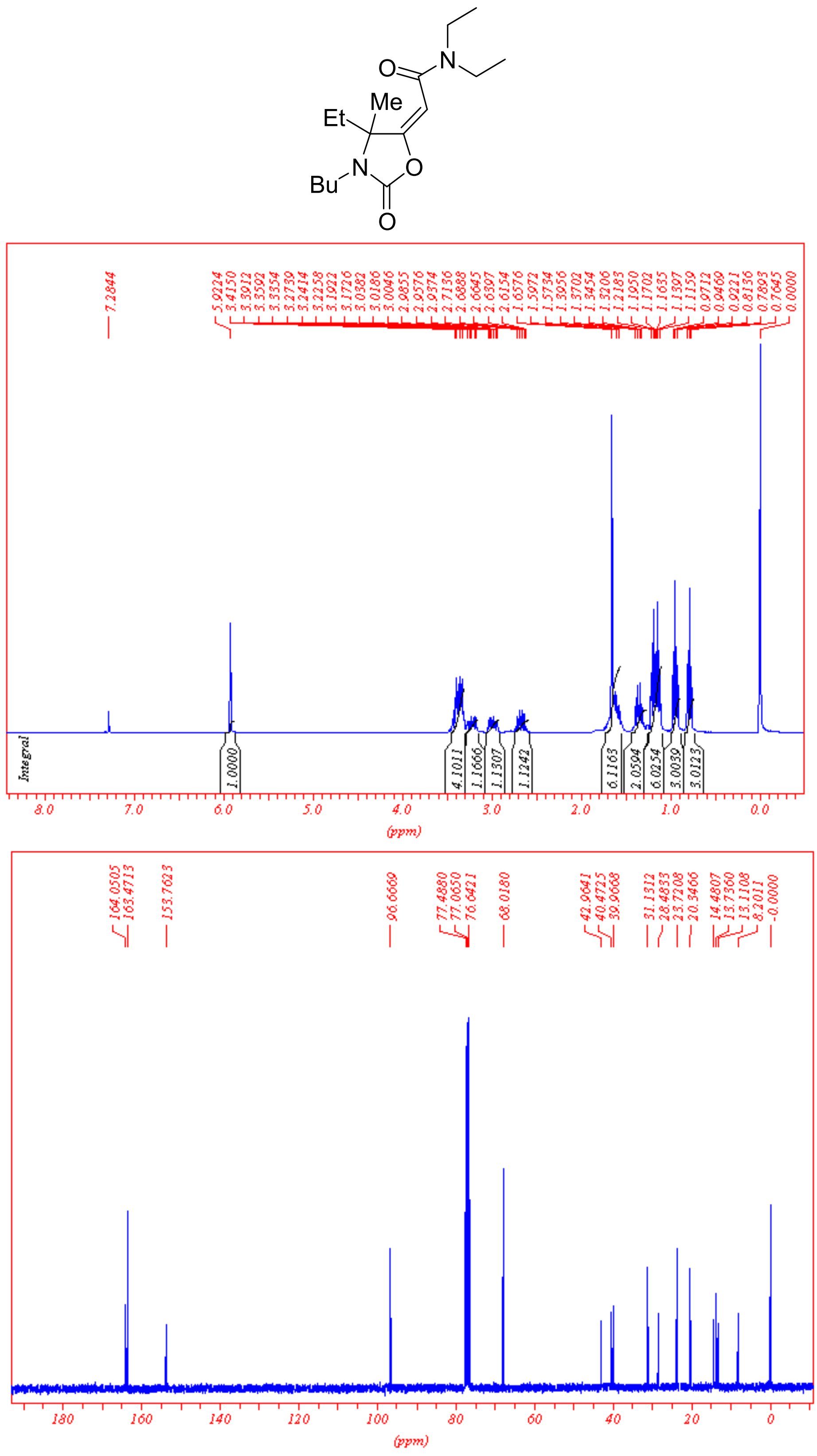

Figure S15. (*E*)-2-(3-Butyl-4-ethyl-4-methyl-2-oxo-oxazolidin-5-ylidene)-*N*,*N*-diethylacetamide (**3dd**-*E*).

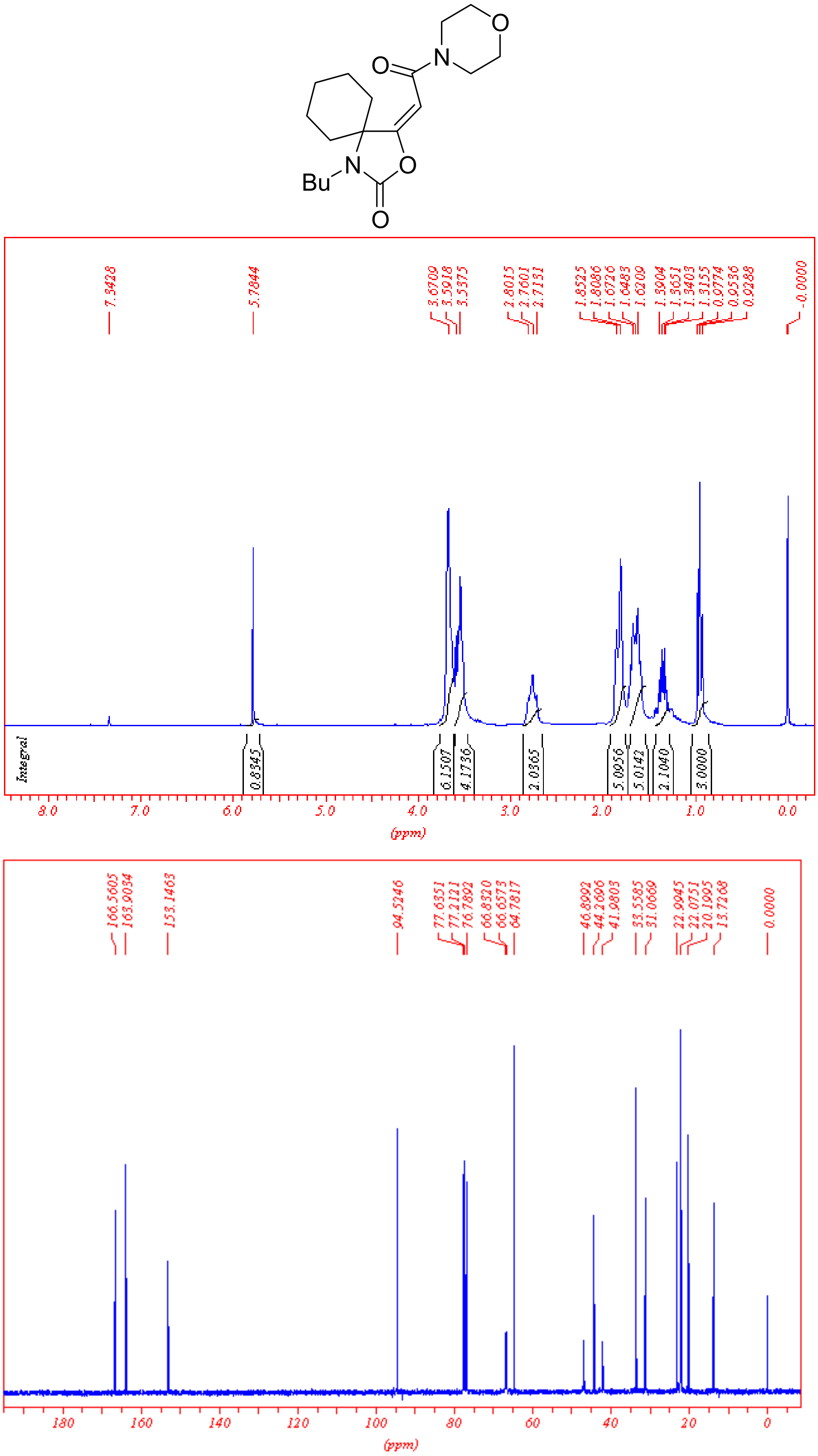

Figure S16. (*E*)-1-Butyl-4-(2-morpholin-4-yl-2-oxoethylidene)-3-oxa-1-azaspiro[4.5]decan-2-one (**3ea**-**E**).

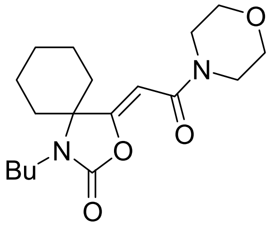

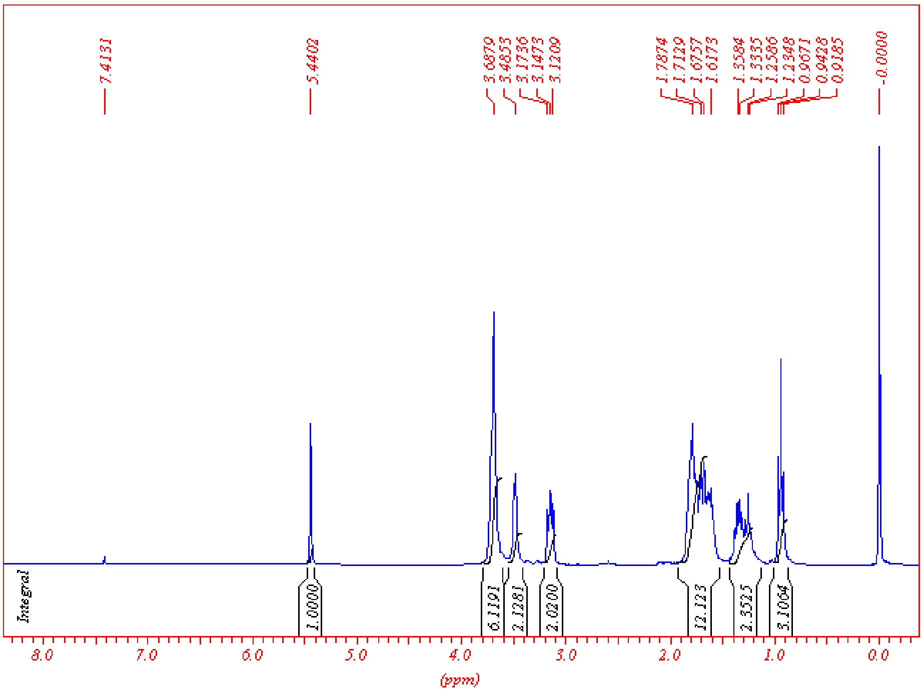

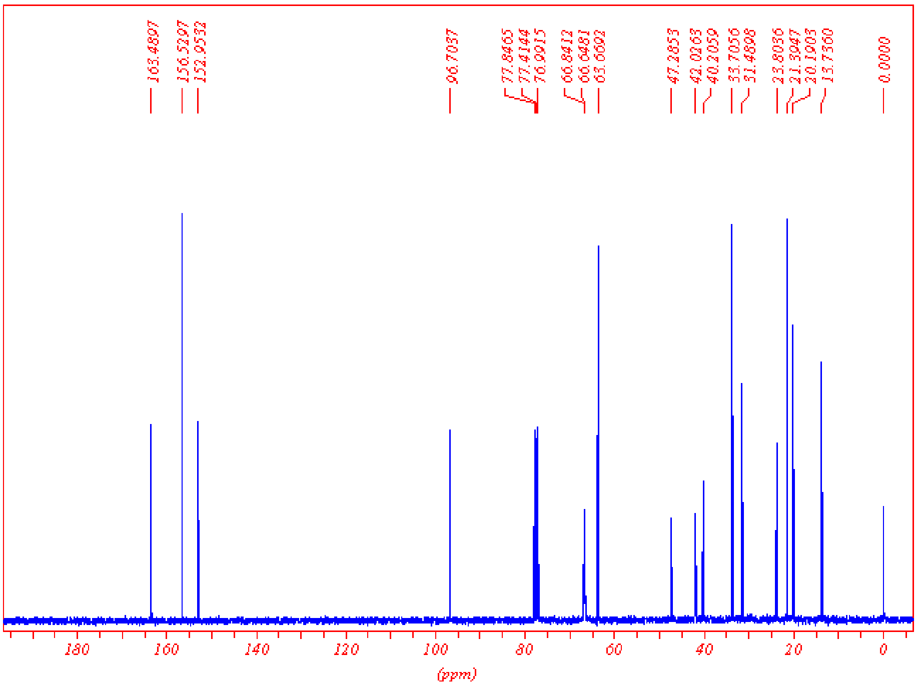

Figure S17. (*Z*)-1-Butyl-4-(2-morpholin-4-yl-2-oxoethylidene)-3-oxa-1-azaspiro[4.5]decan-2-one (**3ea**-*Z*).
